# Analysis of clinical characteristics and risk factors for *Staphylococcus aureus* disseminated infection secondary to acute osteoarticular infections in children

**DOI:** 10.1186/s13052-025-02007-6

**Published:** 2025-05-28

**Authors:** Yingtie Cui, Shiguang Feng, Pengyuan Luo, Zhen Mao, Xiaokang Zhou, Yunzhen Zhang

**Affiliations:** 1 Department of Pediatric Orthopedics, Hebei Children’s Hospital, Shijiazhuang, Hebei China; 2 Department of Orthopaedics, The Ninth Hospital of Xingtai, Xingtai, Hebei China; 3 Department of Plastic Surgery, Hebei Children’s Hospital, No.133 Jianhua South Street, Shijiazhuang, 050000 Hebei China

**Keywords:** Disseminated infection, *Staphylococcus aureus*, Osteoarticular infection, Osteomyelitis, Children

## Abstract

**Background:**

The aim of this study was to investigate the risk factors associated with *Staphylococcus aureus* (*S. aureus*) disseminated infection (DSAI) that occurs secondarily to acute osteoarticular infections (OAI) in children.

**Methods:**

A retrospective analysis of 131 pediatric patients with acute OAI (July 2012–March 2024) was conducted. Patients were categorized into a DSAI group (33 cases) and a non-DSAI group (98 cases). Data analyzed included age, gender, pediatric intensive care unit (PICU) admission, surgical delay, initial symptoms, highest pre-hospital fever, inflammatory markers, pathogen type (MSSA/MRSA), bacteremia, antibiotic duration, postoperative fever length, surgeries (≥ 2), hospital stay, and prognosis.

**Results:**

DSAI primarily affected the lungs, brain, and thorax, with femur and hip joints being the most involved OAI sites. Fever (45.45%) and limb swelling/pain (42.42%) were common symptoms. The DSAI group showed significantly higher CRP levels, bacteremia incidence, MRSA infections, PICU admissions, surgical delays, ≥ 2 surgeries, longer postoperative fever, prolonged hospital stays, and worse prognosis (*P* < 0.05). No significant differences were found in age, gender, pre-admission time, initial symptoms, highest fever, WBC count, ESR, antibiotic duration, or neutrophil percentage (*P* > 0.05). Logistic regression identified bacteremia (OR: 32.232, 95% CI: [2.558-406.068], *P* = 0.007), CRP > 162.375 mg/L (OR: 7.499, 95% CI: [2.044–27.513], *P* = 0.002), and surgical delay > 9.50 days (OR: 7.462, 95% CI: [1.828–30.459], *P* = 0.005) as independent risk factors.

**Conclusion:**

DSAI complicates OAI, leading to a severe course and poor prognosis. High vigilance and early intervention are crucial for pediatric patients with these risk factors.

**Supplementary Information:**

The online version contains supplementary material available at 10.1186/s13052-025-02007-6.

## Introduction

Osteoarticular infections (OAI), which cover acute hematogenous osteomyelitis and suppurative arthritis, are prevalent infectious diseases among children. In developed countries, around 80 in every 100,000 children are afflicted with osteoarticular infections annually [1]. However, in developing countries, the incidence rate is much higher, amounting to as many as 200 in every 100,000 children [2]. Among approximately 7 − 14.1% of children with osteoarticular infections, bacteria can disseminate via the bloodstream and affect other systems, thereby giving rise to conditions like pneumonia, empyema, encephalitis, endocarditis, deep vein thrombosis (DVT), and skin/septic pulmonary embolism, among others [3, 4].

*Staphylococcus aureus* (*S. aureus*) serves as the principal pathogen responsible for osteoarticular infections in children, making up 70–90% of the pathogenic bacteria in such infections [5, 6]. Thanks to its rather strong invasive nature [7], it can trigger disseminated *S. aureus* infection (DSAI) via blood dissemination [8]. When children’s acute OAI are accompanied by secondary DSAI, they frequently encounter a higher mortality rate [9]. Meanwhile, the risk of developing osteoarticular sequelae, such as pathological fractures, limb deformities, and joint stiffness, among others, will also escalate. Early and prompt diagnosis as well as the implementation of corresponding treatment measures are crucial for reducing complications and enhancing prognosis [8]. Nevertheless, in the early stage of DSAI infection, there may be no conspicuous symptoms [10], which poses numerous difficulties for accurate identification at the initial phase [11].

At present, the research specifically centered around DSAI that occurs secondarily to acute OAI is rather limited. There are merely a handful of case reports and cross-sectional studies involving a small number of cases. When it comes to preventing the occurrence of DSAI and its treatment, there is a dearth of essential data to back them up. Against this backdrop, this paper is intended to conduct an in-depth exploration of the diverse risk factors for DSAI secondary to OAI in children. We anticipate that the findings derived from this study can offer valuable assistance for the prevention, early detection, and subsequent treatment of DSAI secondary to acute OAI in children.

## Materials and methods

### Research subjects

**Fig. 1:**
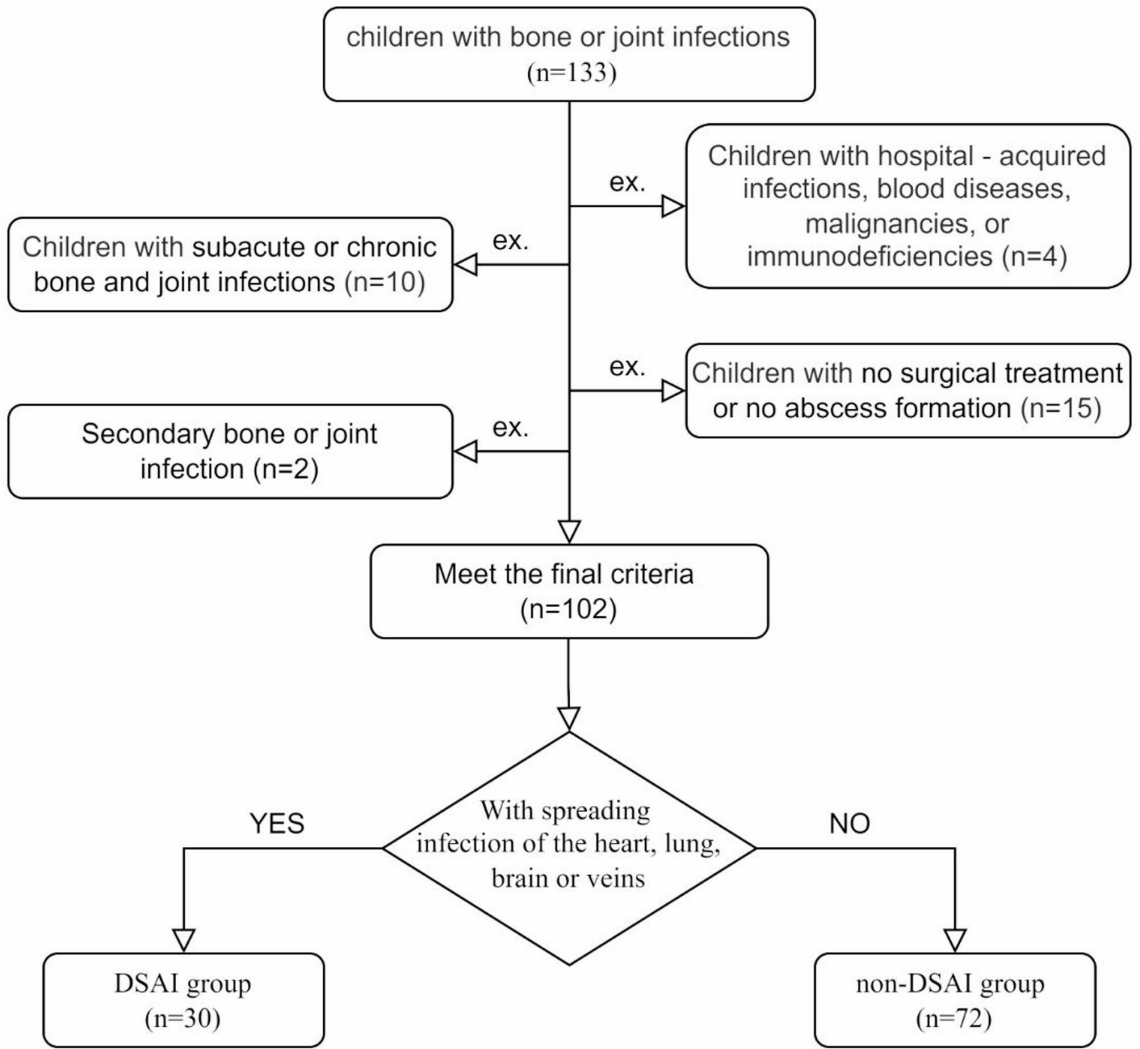
Patient inclusion and exclusion flow chart

This retrospective study encompassed 131 children who were admitted to the Orthopedics Department of Hebei Children’s Hospital and diagnosed with acute OAI during the period from July 2012 to March 2024. Based on whether they developed secondary DSAI, these children were classified into the DSAI group (33 cases) and the non-DSAI group (98 cases). The procedure for patient enrollment is depicted in Fig. 1. Information related to the children’s baseline characteristics, details of the treatment process, and their prognosis was gathered.

This study had received the approval of the Ethics Committee of Hebei Children’s Hospital (No. 2025002). The research plan conformed to the Declaration of Helsinki, and retrospective studies have applied for exemptions from informed consent.

### Inclusion and exclusion criteria

#### Inclusion criteria

① Conforming to the diagnostic criteria for acute OAI [12], that is, the duration of symptoms should be less than 2 weeks.
② Upon admission, magnetic resonance imaging (MRI) reveals anomalies in the osteoarticular area, and the specimens obtained during the surgical procedure are verified through pathological examination.
④ The bacterial culture of the pus obtained during the operation indicates the presence of *S. aureus*.
⑤ Inflammatory indicators are acquired within 72 h following admission.
⑥ The age of the subjects ranges from 1 month to 14 years old.
⑦ All the pathogenic bacteria are community-acquired *S. aureus* infections.
⑧ The guardians have consented to the children’s participation in the study.

#### Exclusion criteria

① Children who also have blood disorders, malignant tumors, or immunodeficiency conditions.
② Children suffering from subacute or chronic osteomyelitis.
③ Children with osteomyelitis without abscess formation who have not received surgical treatment.
④ Children with hospital-acquired *S. aureus* infections.
⑤ Children whose initial site of infection is not the osteoarticular region.
⑥ Children with incomplete clinical data.

#### Management after admission

#### Determination of the primary focus

After the child was admitted to the hospital, clinicians meticulously collected the medical history and accurately located the starting site of the lesion based on the initial clinical manifestations. If the initial symptoms presented as limb swelling, pain, accompanied by functional limitations, the primary focus was mostly considered to be osteoarticular infection.

#### Identification of secondary infection foci

If, following symptoms such as limb swelling, pain, and functional impairment, secondary respiratory symptoms, signs of pericardial tamponade, manifestations of intracranial infection, or persistent high fever occurred, immediately after admission, chest X - ray or computed tomography (CT), echocardiography or pericardiocentesis, cranial MRI, and relevant cerebrospinal fluid tests were carried out to clarify whether there was secondary infection. Bact /ALERT 3D automatic blood culture instrument manufactured by BD Company of the United States was used for bacterial culture, and Bact /ALERT 3D automatic bacterial identification instrument manufactured by BD Company of the United States was used for identification.

#### Comprehensive treatment strategy

After admission, for the two groups of patients, a multi - dimensional comprehensive treatment plan covering antibiotic therapy, surgical intervention, and adjuvant treatment was implemented according to the individualized condition.

#### Antibiotic therapy

In terms of the application of antibacterial drugs, at the beginning of the child’s admission, based on clinical experience, antibiotics targeting Gram - negative and Gram - positive bacteria were selected for combined treatment. Subsequently, the types of antibiotics were adjusted dynamically according to the results of drug sensitivity tests. For infections caused by methicillin - resistant *S. aureus* (MRSA), vancomycin, teicoplanin, or linezolid were preferentially selected. For children with disseminated infections caused by methicillin - sensitive *S. aureus* (MSSA), vancomycin, teicoplanin, or linezolid were initially administered intravenously. After 2–3 weeks of continuous use, according to the severity of the condition and the sensitivity of the pathogen, step - down therapy was implemented, and cefathiamidine or oxacillin sodium was selected for intravenous drip. For children with non - disseminated MSSA infections, according to the drug sensitivity results, first - generation cephalosporins such as ceftezole sodium or cefathiamidine were given intravenously. If the curative effect of the above - mentioned antibiotics did not meet expectations, rifampicin tablets or compound sulfamethoxazole tablets were added for oral administration. Intravenous antibacterial drugs were continuously used until the child’s body temperature and C - reactive protein (CRP) level returned to the normal range. Subsequently, the treatment was switched to oral antibiotics for a further 2 - week continuation.

#### Surgical intervention

For all children, if MRI indicated the presence of intramedullary abscess, subperiosteal abscess, joint effusion, or purulent fluid was obtained by puncture, incision and drainage, or a combination of cortical bone fenestration and decompression with continuous irrigation and drainage was performed. The indication for removing the drainage tube was that the drainage fluid was clear and transparent and the child’s body temperature returned to normal. For children with pericarditis, pericardiectomy combined with abscess debridement was performed. If a child was diagnosed with empyema, closed - chest drainage was performed in a timely manner. After the operation, the affected limb was fixed in the functional position with a brace or plaster splint, and the duration of limb fixation was accurately determined according to the degree of bone destruction shown by X - ray examination.

### Observation indicators and definitions

The following indicators related to the patients were documented:

1. Baseline Information: This encompasses age and gender.
2. Treatment-Related Information: Includes the time elapsed prior to admission, whether the patient was admitted to the pediatric intensive care unit (PICU), the duration of delay in surgical intervention, the initial symptoms presented, and the length of the hospital stay.
3. Condition-Related Information: It involves the highest body temperature prior to hospitalization, Inflammatory markers upon admission (white blood cell (WBC) count, percentage of neutrophils, C-reactive protein level, erythrocyte sedimentation rate), pathogens (either MSSA or MRSA), the occurrence of bacteremia(Blood culture was taken when temperature ≥ 38.5℃), the duration of using sensitive antibiotics, the duration of postoperative fever, as well as ≥ 2 operations.
4. Prognosis Information: Patients were followed up for 1 year postoperatively. If pathological fracture, limb deformity or other adverse prognostic events occurred during the follow-up period, the monitoring was continued; however, follow-up was discontinued at 1 year after surgery for those without any adverse prognosis.

### Related definitions

Bacteremia: It is defined as the identification of *S. aureus* in blood cultures, with the clinical course aligning with that of an *S. aureus* infection [10].

Multifocal Infection: This is characterized by the involvement of two or more non-contiguous bones and/or joints.

Disseminated Infection: It pertains to the presence of multifocal infection, DVT, pneumonia, empyema, encephalitis, pulmonary or skin septic embolism, and/or pericarditis, among other manifestations [4, 7, 11].

Delay in Operation Time: This refers to the interval from the onset of symptoms associated with acute OAI to the time of surgical intervention.

≥ 2 Operations: It indicates that two or more surgical treatments were carried out when an abscess reformed either in the same or different anatomical locations.

Duration of Postoperative Fever: It represents the length of time it takes for the postoperative body temperature to drop to 37 °C.

Positive Pus Culture: This implies that pathogens were cultured from the specimens collected through subperiosteal or joint puncture before the operation and/or from those obtained during the surgical procedure.

Complicated Course: This includes admission to the PICU, Delay in Operation Time, ≥ 2 Operations, the length of the hospital stay, and the duration of postoperative fever.

Duration of Using Sensitive Antibiotics: It refers to the time span from the appearance of the initial symptoms to the commencement of the use of sensitive antibiotics (as determined by the results of culture and drug sensitivity testing).

### Statistical analysis

All the data collected in this study were analyzed using SPSS 26.0 software. Normally distributed measurement data were expressed as mean ± standard deviation (SD), while non-normally distributed measurement data were expressed as median (interquartil range), and the comparisons were examined by Student-t test and Mann-Whitney test (non parametric distribution). The categorical data were expressed as n(%), and the differences between the two groups were examined by chi-square analysis or Fisher’s exact test. The receiver operating characteristic (ROC) curve was used to evaluate the diagnostic efficacy of different indicators for DSAI. A binary logistic regression analysis on the factors that were statistically significant in the univariate analysis, and were presented with the odds ratios (ORs) and their 95% confidence intervals (CIs). *P*<0.05 was considered statistically significant.

## Results

### Baseline information and case characteristics of children with DSAI

**Fig. 2:**
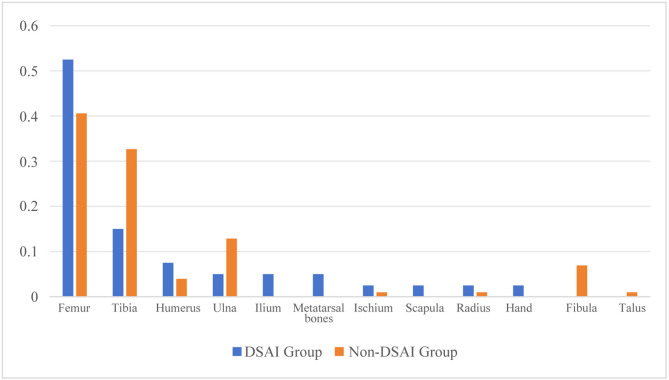
Percentage of Infected Skeletal Sites in Two Groups

**Fig. 3:**
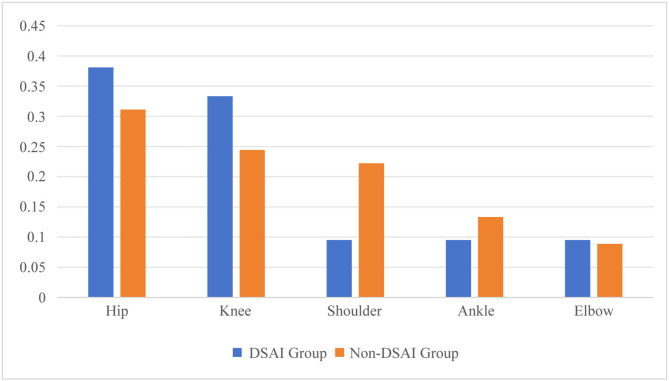
Percentage of Infected Joint Sites in Two Groups

Among the two groups of children, the femur was the most prone to dissemination in primary bone infections, accounting for 52.50% (Fig. 2). In primary joint infections, the hip joint and knee joint were the most prone to dissemination, accounting for 38.10% and 33.33% respectively (Fig. 3). Among the 33 children diagnosed with DSAI, 20 were male, accounting for 60.61% (20/33), while 13 were female, making up 39.33% (13/33). The male-to-female ratio was 1.54:1. Their ages spanned from 1.83 to 168 months, with a median age of 61.87 months. Specifically, 15 children were under 3 years old, constituting 45.45% of the total number, and 13 children were aged between 7 and 14 years, accounting for 39.39% of the total. When it came to the parts other than bones and joints affected by DSAI, the lungs were the most frequently involved, with 31 cases (52 sites in total), which accounted for 93.94% (31/33). Next, the brain and the thoracic cavity had 8 and 9 cases, accounting for 24.24% (8/33) and 27.27% (9/33). There were 3 cases in the pericardial cavity, representing 9.09% (3/33). Additionally, 5 cases (15.15%, 5/33) were complicated by lower extremity venous thrombosis (Supplementary Table 1).

### Univariate analysis of the formation of DSAI

The levels of CRP, the incidence of bacteremia, MRSA infection, admission to the PICU, delayed operation time, the occurrence of ≥ 2 operations, postoperative fever duration, length of hospital stay, and poor prognosis in the children in the DSAI group were all higher than those in the non-DSAI group, and the differences were statistically significant (*P* < 0.05). However, there were no statistically significant differences in gender, age, the time elapsed prior to admission, initial symptoms, the highest body temperature before hospitalization, WBC count, erythrocyte sedimentation rate, duration of using sensitive antibiotics, and percentage of neutrophils (*P* > 0.05). **(See** Table 1)

**Table 1:** Comparison of clinical features between non-DSAI group and DSAI group

Variate	DSAI Group(*n* = 33)	Non-DSAI Group(*n* = 98)	t/Z/χ2	*P*
Gender [n(%)]			0.304	0.581
Male	20 (60.61)	54 (55.10)		
Female	13 (39.39)	44 (44.90)		
Age (month)	61.87 (1.83, 168.00)	68.01 (1.00, 180.00)	-0.449	0.654
The time elapsed prior to admission (day)	4.79 (1.00, 11.00)	6.18 (1.00, 30.00)	-1.837	0.066
Initial symptoms [n(%)]			2.598	0.273
Fever	15 (45.45)	29(29.59)		
Swelling pain	14 (42.42)	47 (47.56)		
Swelling pain with fever	4 (12.12)	22(22.45)		
The highest body temperature before hospitalization (℃)	38.82 ± 5.93	39.23 ± 0.81	-0.413	0.682
Delayed operation time (day)	12.55 ± 8.81	8.09 ± 5.196	-3.468	0.001
≥ 2 operations [n(%)]			4.146	0.042
Yes	7 (21.21)	8 (8.16)		
No	26 (78.79)	90 (91.84)		
Admission to the ICU [n(%)]			38.668	< 0.001
Yes	25 (80)	17 (18.06)		
No	8 (20)	81 (81.94)		
Lngth of hospital stay (d)	37.27 ± 12.88	27.49 ± 9.60	-3.996	< 0.001
WBC (10ˆ9/L)	14.49 ± 8.93	15.71 ± 6.81	− 0.778	0.438
NEU (%)	74.59 ± 14.05	69.49 ± 15.01	-1.709	0.09
CRP (mg/L)	180.92 ± 61.15	101.29 ± 63.25	-6.193	< 0.001
ESR (mm/L)	52.21 ± 32.35	63.13 ± 32.31	-1.676	0.099
Time to use sensitive antibiotics (day)	6.06 ± 2.96	7.25 ± 5.37	-1.176	0.242
Postoperative fever duration (day)	10.42 ± 7.36	4.06 ± 4.60	-4.671	< 0.001
MRSA [n(%)]			6.383	0.012
yes	22 (66.67)	40 (40.82)		
no	11 (33.33)	57 (58.16)		
Bacteremia [n(%)]			27.481	< 0.001
Yes	32 (96.97)	44 (44.90)		
No	1 (3.03)	54 (55.10)		
Prognosis [n(%)]			4.528	0.033
Good	21(66.64)	80(81.63)		
Poor	12(36.36)	18(18.37)		

DSAI, Staphylococcus aureus disseminated infection. WBC, White Blood Cell. NEU, Neutrophils. CRP, C-reactive protein. ESR, Erythrocyte Sedimentation Rate. MRSA, Methicillin-Resistant S. aureus

### Prediction of the diagnostic efficacy of DSAI

**Fig. 4:**
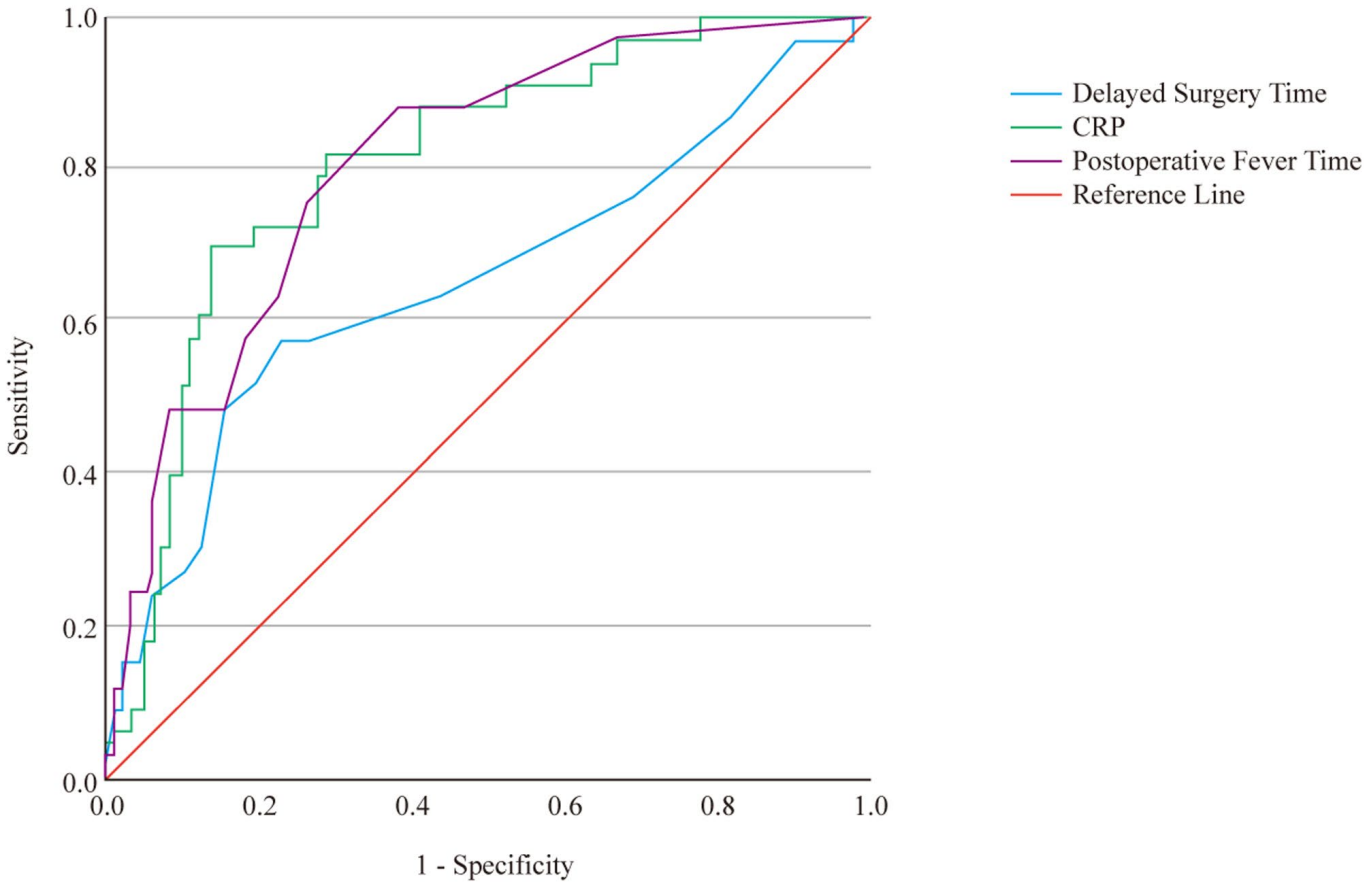
ROC curves of CRP, postoperative fever duration, and delayed operation time for predicting the presence of disseminated infection

Figure 4 shows the ROC curves of three indicators, namely CRP, postoperative fever duration, and delayed operation time, for predicting disseminated infection. All three indicators had relatively good diagnostic efficacy (*P* < 0.05). The optimal cut-off value of CRP was 162.375 mg/L, with a sensitivity of 67.90%, a specificity of 86.70%, an area under the curve (AUC) of 0.809, and a 95%CI of 0.726–0.892. The optimal cut-off value of delayed operation time was 9.50 days, with a sensitivity of 57.60%, a specificity of 77.60%, an AUC of 0.655, and a 95%CI of 0.537–0.773. The optimal cut-off value of postoperative fever duration was 3.50 days, with a sensitivity of 87.9%, a specificity of 62.2%, and an AUC of 0.807, and a 95%CI of 0.725–0.889.

### Analysis of risk factors for DSAI

CRP (> 162.375 mg/L = 1, < 162.375 mg/L = 0), delayed operation time (> 9.50 days = 1, < 9.50 days = 0), postoperative fever duration (> 3.50 days = 1, < 3.50 days = 0), whether admitted to the PICU (yes = 1, no = 0), whether having bacteremia (yes = 1, no = 0), whether having MRSA infection (yes = 1, no = 0) and whether having ≥ 2 operations (yes = 1, no = 0) were incorporated into the logistic regression analysis. Bacteremia (odds ratio (OR): 32.232, 95%CI: [2.558-406.068], *P* = 0.007), CRP > 162.375 mg/L (OR: 7.499, 95% CI: [2.044–27.513], *P* = 0.002), and delayed operation > 9.50 days (OR: 7.462, 95% CI: [1.828–30.459], *P* = 0.005),were high-risk factors for the occurrence of disseminated infection. **(See** Table 2)

**Table 2:** Logistic regression analysis of different indicators predicting disseminated infection

Variate	OR	95% CI	*P*
Admitted to the ICU	3.137	0.801–12.287	0.101
Delayed operation > 9.5d	7.462	1.828–30.459	0.005
CRP>162.375 mg/L	7.499	2.044–27.513	0.002
MRSA	3.961	0.987–16.038	0.054
Bacteremia	32.232	2.558-406.068	0.007
≥ 2 operations	1.565	0.334–7.338	0.570
Postoperative fever >3.5d	4.342	0.834–22.608	0.081

ICU, Intensive Care Unit. CRP, C-reactive protein. MRSA, Methicillin-Resistant S. aureus

## Discussion

*S. aureus* is the most common pathogen causing OAI in children. It can spread to other organs through the bloodstream, resulting in severe infectious complications, such as pneumonia, empyema, endocarditis, osteomyelitis, suppurative arthritis, skin and soft tissue infections, deep abscesses, suppurative thrombophlebitis, etc., leading to a relatively high morbidity and mortality rate, and the prognosis is often poor [13]. The diagnosis and treatment of DSAI secondary to OAI are extremely challenging, mainly because DSAI lacks specific manifestations in clinical and imaging aspects and is easily confused with other infectious or non-infectious diseases. Meanwhile, this study found that there were no significant differences in the initial symptoms at the time of medical treatment and the highest body temperature before hospitalization between the children in the DSAI group and those in the non-DSAI group, which brought difficulties to the early diagnosis of DSAI. Moreover, currently, there is no separate report on DSAI secondary to OAI, but it is only included in the studies on bacteremia. In this study, we explored the relevant factors and clinical characteristics affecting DSAI secondary to OAI in children.

OAI is not uncommon among children. *S. aureus* is one of the important pathogens causing OAI in children [14]. However, there is no fixed, unified and precise value for the specific incidence of its disseminated infection among children with OAI. Previous studies have found that the incidence of DSAI among children is 7–15% [3, 15], which is lower than the incidence of 25.19% in this study. The following reasons are considered: ① Only children with OAI who had abscess formation and underwent surgery were included in this group of cases, and the pathogen was *S. aureus*; ② The age distribution of the included children was different; ③ In different regions and with different medical levels, the early diagnosis and early treatment of the disease vary. The above reasons have led to significant differences in the results of different studies.

We found that the most common metastatic site of disseminated staphylococcal adjacent implant infection (DSAI) caused by osteomyelitis adjacent to implants (OAI) was the lungs, followed by the brain and the thoracic cavity. This is consistent with the research by Clerc Berestein M et al. [16]. They conducted a study on 112 children with metastatic infections caused by *S. aureus* bacteremia (SAB) and believed that children with SAB whose primary focus was OAI were more likely to have pulmonary metastatic infections. The main reason is considered to be that after the pathogen enters the bloodstream, it flows back to the pulmonary circulation along with the venous system, and then the pathogen colonizes in the lungs, resulting in infection.

CRP is an acute-phase reactant protein. Its level will rise rapidly in cases of infection, inflammation, tissue injury and other conditions. Its level is closely related to the severity of the infection and can be regarded as one of the important indicators for judging the prognosis of infection [17]. This study found that the CRP level of children in the DSAI group was significantly higher than that in the non-DSAI group, and CRP > 162.375 mg/L was an independent risk factor for DSAI. Therefore, for children with OAI who have a relatively high CRP level, great vigilance should be paid to the possibility of DSAI.

Bacteremia refers to a systemic infection caused by pathogenic bacteria invading the bloodstream, growing and multiplying in the bloodstream, and producing toxins. Once bacteremia occurs, the pathogenic bacteria can spread to various organs throughout the body along with the bloodstream, resulting in severe infectious complications [18]. In this study, the incidence of bacteremia in the children in the DSAI group was significantly higher than that in the non-DSAI group, and bacteremia was an independent risk factor for DSAI. It is generally believed that acute OAI is the result of transient bacteremia with metastatic infection. Some studies have shown that the negative rate of blood culture in OAI is 26.6% [4], while the negative rate of blood culture upon admission in this study was 39%, further confirming that bacteremia is transient. And a positive bacterial culture upon admission indicates that the duration of bacteremia is relatively long. Moreover, the longer the duration of bacteremia, the greater the possibility of developing DSAI. Clerc Berestein M, et al. [16] found in their study on metastatic infections caused by bacteremia that a positive blood culture persisting for more than 48 h after the first blood culture increased the possibility of metastatic infection by three times. Therefore, for children with OAI, blood culture examinations should be carried out in a timely manner so as to detect bacteremia early and provide active treatment.

Surgical treatment and antibiotic treatment are important primary treatments for OAI. The purpose of surgery is to remove the focus of infection, drain pus, relieve the inflammatory response and promote the recovery of the condition. The choice of the timing of surgery is crucial for the treatment effect and prognosis. In this study, the delayed operation time of children in the DSAI group was significantly longer than that in the non-DSAI group, and a delayed operation time > 9.50 days was an independent risk factor for DSAI. If the operation is delayed for too long, it may lead to the spread of infection, the aggravation of the condition and an increased risk of DSAI. Therefore, for children with OAI, surgical treatment should be carried out as early as possible to reduce the risk of DSAI. Regarding the use of antibiotics in children with OAI, previous studies [10] found that the predictive factor related to the occurrence of metastatic infections (including single-site infections and DSAI) in children with bacteremia was a 48-hour delay in appropriate antibacterial treatment. The results of this study showed that there was no difference in the duration of using sensitive antibiotics between the two groups. However, this does not mean a decline in the effect of antibiotic treatment. It is mainly considered that timely surgical removal of the source of infection has a more obvious effect on the prognosis.

Among *S. aureus* strains, MRSA has become an important issue of global concern [19]. The presence of MRSA biofilms in osteomyelitis has brought additional challenges to treatment [20]. Joint infections caused by MRSA often have a more complicated course (including faster progression of the disease, longer hospital stays and more surgeries) and a poorer prognosis [21, 22]. Currently, there are controversies in reports on the occurrence of DSAI in bacteremia caused by pathogenic bacteria being MRSA. Some reports suggest that MRSA and delayed antibiotic treatment are related to the occurrence of metastatic infections (including DSAI) in multivariate models [23, 24]. Le J, et al. [25] believe that when the pathogenic microorganism is MRSA, DSAI is more common among children with bacteremia (including those with OAI as the origin). This is consistent with our results. In this study, it was found that the incidence of the pathogenic microorganism MRSA in the DSAI group was significantly higher. However, in the logistic regression analysis, OAI caused by MRSA had no significant correlation with disseminated infection. We think that the main reason is that there was no difference in the time of using sensitive antibiotics at the beginning of the onset between the two groups of cases, and the disseminated group did not delay the use of effective antibiotics [16].

Previous studies on children with OAI having a complicated disease course have shown that the following reasons are more likely to lead to a complicated disease course, including bacteremia (MRSA infection), combined with venous thrombosis, combined with DSAI. A complicated disease course will result in longer hospital stays, more surgical interventions and more frequent visits to the PICU [24–27]. This is similar to the results of our study. The results of this study showed that the incidences of PICU admission, ≥ 2 operations, postoperative fever duration, length of hospital stay and poor prognosis in the children in the DSAI group were all significantly higher than those in the non-DSAI group. However, in the multivariate analysis, these factors were not independent risk factors for DSAI. This may be because these factors have a certain correlation with the occurrence of DSAI, but they may be the results rather than the causes of DSAI. For example, children with DSAI are often in a more serious condition and need to be admitted to the PICU for monitoring and treatment. They may also need to undergo multiple surgeries, and the postoperative fever duration and length of hospital stay will be correspondingly prolonged, and the incidence of poor prognosis will also increase. Some studies believe that the presence of disseminated infection is related to the long-term prognosis of OAI [4]. The results of this study are consistent with this, and the DSAI group has a poorer prognosis, with an incidence of long-term complications of approximately 36.36%.

This study has certain limitations. First, this study is a single-center retrospective study with a relatively small sample size, which may lead to selection bias and information bias. Secondly, some cases of OAI only underwent chest X-ray examinations instead of CT. Some mild disseminated metastatic lesions were likely to be missed, and the actual prevalence of DSAI among children with OAI might have been underestimated. In addition, this study did not analyze factors such as the immune status and gene polymorphism of the children, and these factors might also be related to the occurrence of DSAI. Therefore, in the future, it is necessary to conduct multi-center, large-sample, prospective studies to further explore the risk factors for DSAI secondary to OAI in children, so as to provide a more powerful basis for clinical diagnosis and treatment.

## Conclusion

In summary, this study has found that bacteremia, CRP > 162.375 mg/L, and delayed operation time > 9.50 days are independent risk factors for DSAI secondary to OAI in children. For children with these risk factors, great vigilance should be paid to the possibility of DSAI. Relevant examinations should be carried out in a timely manner so as to achieve early detection and early treatment and improve the prognosis of children.

## Electronic supplementary material

Below is the link to the electronic supplementary material.

Supplementary Material 1

## Data Availability

Not applicable.

## Abbreviations

CIs: Confidence intervals
CRP: C - reactive protein
CT: Computed tomography
DSAI: Staphylococcus aureus disseminated infection
DVT: Deep vein thrombosis
MRI: Magnetic resonance imaging
MRSA: Methicillin-resistant staphylococcus aureus
MSSA: Methicillin-ausceptible staphylococcus aureus
OAI: Acute osteoarticular infections
ORs: Odds ratios
ROC: Receiver operating characteristic
S. aureus: Staphylococcus aureus
SAB: S. aureus bacteremia
SD: Standard deviation
WBC: White blood cell
