# Supplementary material for: Analysis of clinical characteristics and risk factors for Staphylococcus aureus disseminated infection secondary to acute osteoarticular infections in children
Source: Ital J Pediatr. 2025 May 28;51:160. doi: 10.1186/s13052-025-02007-6 (PMC12121166; doi:10.1186/s13052-025-02007-6)
Supplement: Supplementary file 1 — Supplementary Material 1 [file 13052_2025_2007_MOESM1_ESM.docx]

Supplmentary table 1 Characteristic on computer tomography.

| Group | Computer tomography | Case (%) |
| --- | --- | --- |
| Lung |  | 30 |
|  | Bilateral | 30（100%） |
|  | Multiple | 30（100%） |
|  | scattered | 30（100%） |
|  | Subpleural | 30（100%） |
|  | Nodule | 26（86.67%） |
|  | Agglomerate | 22（73.33%） |
|  | Obscure boundary | 23（76.67%） |
|  | Heterogeneity of density | 24（80%） |
|  | Ballonet | 24（80%） |
|  | Lung abscess | 14/（46.67%） |
|  | Cavity | 14/（46.67%） |
|  | Consolidation | 15（50%） |
|  | Necrosis | 16（53.33%） |
|  | Gas-fluid level | 9（30%） |
|  | Pleural thickening | 29（96.67%） |
|  | Pleural effusion | 21（70%） |
|  | Soft tissue of chest wall | 7（23.33%） |
| Cerebrum | Broadening | 8 |
|  | Sulus | 5（62.5%） |
|  | Ventricle | 5（62.5%） |
|  | Double frontotemporal extracerebral space | 7（87.5%%） |
|  | Subtentorial space | 2（25%） |
|  | Cisterna magna | 4（50%） |
| Pericardium |  |  |
|  | Pericardial effusion | 3（9.09%） |

There were 33 patients with disseminated infection, 32 of whom underwent chest CT examination and 1 of whom did not. Among them, there were a total of 30 children with pulmonary infection. The main manifestations were multiple nodules/clump-like lesions scattered in both lungs, mainly under the chest model. Some of the children had small air sac cavities, consolidation, necrosis, voids, and liquid air plane to varying degrees. Pleural effusion occurred in 70% of the children, of which 9 cases had more pleural effusion, closed thoracic drainage was performed, and 12 cases had a small amount of pleural effusion without any treatment. The brain widened in different parts of 8 children, 8 children with pericardial effusion, 3 of which had a large amount of effusion and symptoms of pericardial tamponade and received surgical treatment, 5 cases with a small amount of effusion, no symptoms and no treatment.
